# Chirality Induction through Nano‐Phase Separation: Alternating Network Gyroid Phase by Thermotropic Self‐Assembly of X‐Shaped Bolapolyphiles

**DOI:** 10.1002/anie.201911245

**Published:** 2020-01-07

**Authors:** Changlong Chen, Robert Kieffer, Helgard Ebert, Marko Prehm, Rui‐bin Zhang, Xiangbing Zeng, Feng Liu, Goran Ungar, Carsten Tschierske

**Affiliations:** ^1^ State Key Laboratory for Mechanical Behaviour of Materials Shaanxi International Research Center for Soft Matter Xi'an Jiaotong University Xi'an 710049 P. R. China; ^2^ Institute of Chemistry Martin-Luther-University Halle-Wittenberg Kurt-Mothes-Straße 2 06120 Halle Germany; ^3^ Department of Materials Science and Engineering University of Sheffield Sheffield S1 3JD UK; ^4^ Department of Physics Zhejiang Sci-Tech University Hangzhou 310018 P. R. China

**Keywords:** chirality, liquid crystals, mirror symmetry breaking, single gyroid, soft matter

## Abstract

The single gyroid phase as well as the alternating double network gyroid, composed of two alternating single gyroid networks, hold a significant place in ordered nanoscale morphologies for their potential applications as photonic crystals, metamaterials and templates for porous ceramics and metals. Here, we report the first alternating network cubic liquid crystals. They form through self‐assembly of X‐shaped polyphiles, where glycerol‐capped terphenyl rods lie on the gyroid surface while semiperfluorinated and aliphatic side‐chains fill their respective separate channel networks. This new self‐assembly mode can be considered as a two‐color symmetry‐broken double gyroid morphology, providing a tailored way to fabricate novel chiral structures with sub‐10 nm periodicities using achiral compounds.

## Graphical abstract

**Self‐sorting** of the two poorly compatible side‐chains of X‐shaped polyphiles into their own networks gives rise to a chiral gyroid cubic phase (the “single gyroid” *I*4_1_32) with two chemically non‐equal networks. This is the first alternating network gyroid cubic liquid crystal, and the first liquid crystal with chirality solely based on phase separation, allowing the self‐assembly of chiral structures with sub‐10 nm periodicities.

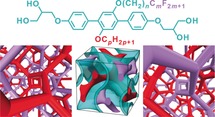

New routes to chirality from initially achiral systems are of particular contemporary interest for obtaining chiral templates in asymmetric synthesis and catalysis.^1^ This is important for the use in different fields of material‐ and nano‐science^2^ as well as for the understanding of fundamental principles of the emergence of biological homochirality.^3^ Creating chirality in liquids and liquid crystals (LCs), having no fixed positions of individual molecules, is especially challenging.^4^, ^5^ Nevertheless, it was recently achieved by mirror symmetry breaking through synchronization and locking‐in of transient chiral conformations and configuration.^6^ Here we report a new approach to spontaneous generation of chirality based on nano‐phase segregation. In the reported case, breaking the inherent mirror symmetry of the double gyroid cubic phase ($Ia\bar{3}d$ , Q230), known from lyotropic^7^ and thermotropic liquid crystals (LCs)^8^ (Figure 1 a), is achieved by self‐assembly of X‐shaped polyphilic molecules with two different chains at opposite sides of a rod‐like molecular core.8b The cores organize along the gyroid minimal surface, forming a wall that separates the two enantiomeric infinite networks involving these chains. Nano‐phase separation of the two poorly compatible semiperfluorinated and aliphatic side‐chains, into their own networks (blue and red in Figure 1 b), gives rise to a gyroid cubic phase with two chemically non‐equal networks (the “single gyroid” *I*4_1_32, Q214). This structure has broken mirror symmetry and represents the first alternating network gyroid cubic LC, and the first LC with chirality solely based on phase separation. Previous attempts to produce a single gyroid structure were based on replication from butterfly wings,^9^ lithography^10^ and templating.^11^ The alternating double network gyroid was found in narrow composition ranges of multiblock copolymer blends,^11^, ^12^ leading to structures in the >100 nm range in all cases. The new concept reported herein provides a tailored way to fabricate chiral structures with much smaller sub‐10 nm periodicities, which are of great potential in nano‐templating and as enantiospecific membranes for use in enantiomer separation.

**Figure 1:**
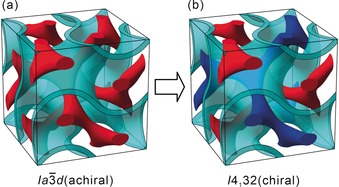
a) The double‐network gyroid $Ia\bar{3}d$ cubic phase and b) the alternating double gyroid cubic phase involving two networks comprising different components separated by the G minimal surface (this work).

To this end, a series of X‐shaped molecules has been specifically designed and synthesized. These molecules are based on a *p*‐terphenyl core terminated by two hydrogen‐bonded polar glycerol groups, and bearing two laterally attached incompatible chains, that is, an aliphatic hydrocarbon chain (R_H_) and a semiperfluorinated chain (R_F_) containing a short ‐(CH_2_)_*n*_‐ spacer and a long perfluorinated end segment ‐C_*m*_F_2*m*+1_ (see Table 1). For details on synthesis and experimental procedures, see the Supporting Information. Most compounds form enantiotropic LC phases, except for compound **1 c,** which only exhibits a monotropic soft crystal phase (see Table 1). Here, compounds **1 b**, **2** and **3** are of main focus, all forming a novel alternating double network gyroid phase with lattice parameters below 10 nm upon both heating and cooling.

**Table 1:** Structure information, phase transitions (transition temperatures and associated enthalpy changes), and lattice parameters of X‐shaped molecules **1**—**3**. 
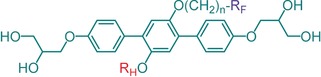

Comp.	R_H_	*n*	R_F_	Phase transitions^[a]^	Lattice parameter [nm]^[b]^	Volume fraction of side chains^[c]^ [%]
**1 a**	‐C_20_H_41_	6	‐C_8_F_17_	Cr_1_ [18] Cr_2_ [55] Col_hex_/*p*6*mm* [81] iso *1.1* ^*^ *4.2* ^*^ *2.5*	6.89	62.7
**1 b**	‐C_20_H_41_	6	‐C_10_F_21_	G [24] Cub/*I*4_1_32 [88] iso *– 2.0*	9.19	64.5
**1 c**	‐C_20_H_41_	6	‐C_12_F_25_	Cr [106] (Cr_Lam_ [103]) iso *2.0* ^*^ *11.5*	6.32	66.2
**2**	‐C_20_H_41_	4	‐C_12_F_25_	Cr [104] Cub/*I*4_1_32 [110] iso *11.4* *1.9*	8.70	64.9
**3**	‐C_22_H_45_	4	‐C_10_F_21_	Cr [35] Cub/*I*4_1_32 [102] iso *16.0* *2.4*	8.88	64.0

[a] Recorded from first DSC heating at 10 K min^−1^ (see Figure S1) and POM; brackets mean metastable phase (only observed upon heating); transition temperatures *T* (°C) are given in square brackets, associated enthalpy changes Δ*H* (kJ mol^−1^) are given in lower lines in italics; [b] Determined by synchrotron powder small angle X‐ray scattering; [c] Volume fraction of side chains measured using Material Studio. Abbreviations: Cr, Cr_1_, Cr_2_=crystalline solid; G=glassy solid; Col_hex_/*p*6*mm*=Hexagonal columnar phase with *p*6*mm* symmetry; Cub/*I*4_1_32=alternating double network gyroid cubic phase with *I*4_1_32 symmetry; Cr_Lam_=lamellar soft crystal phase; Iso=isotropic liquid. * Partial crystallization.

As the length of the perfluorinated side chain is increased, compounds **1 a**–**c** exhibit a phase sequence Col_hex_/*p*6*mm*—Cub/*I*4_1_32—Cr_Lam_ (Table 1 and Figure 2 a–c). Compound **1 a**, bearing the shortest semiperfluorinated chain, is found to form a hexagonal columnar phase showing a birefringent fan‐like texture interspersed with black homeotropic regions (Figure S2a, Supporting Information). The sharp Bragg reflections in small angle X‐ray scattering (SAXS) are indexable on a 2D hexagonal lattice with unit cell parameter *a*_hex_=6.89 nm, showing only diffuse scattering in the wide angle region, characteristic of a LC (Figures 2 a, S3 and Tables 1, S1). The lattice parameter corresponds to about three times the molecular length (*L*_mol_=2.3–2.6 nm measured between the two terminal polar groups). The electron density map reconstructed based on *p*6*mm* symmetry (Figure S10) shows a partly segregated two‐color tiling composed of a lower‐density (alkyl) column and two higher density (mixed) columns.^13^ The analysis of the two‐color Col_hex_ phase is described in Section 5 of the Supporting Information. The aromatic cores make up the walls between the columns with glycerol groups forming the hydrogen bonding networks at cell edges (Figure 4 g).

**Figure 2:**
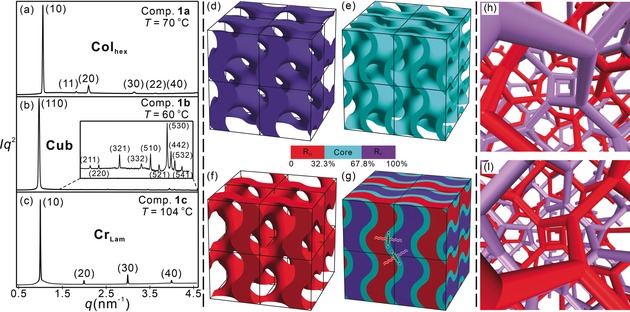
a–c) Synchrotron SAXS diffractograms of the mesophases of compounds **1 a**–**c** at given temperatures. d–g) Reconstructed electron density maps in 2×2×2 unit cell. The boundaries are estimated by the volume fractions of each segment: d) network formed by semiperfluorinated chains; e)  gyroid minimal surface formed by molecular cores; f) network composed of the aliphatic chains; g) overall view of the electron density map (purple=perfluorinated chains, cyan=rod‐like cores of aromatic and polar segments, red=alkyl chains). h,i) Views along a right‐handed (h) and a left‐handed (i) 4_1_ screw axis of the Cub_bi_/*I*4_1_32 phase, illustrating the opposite chirality of the two networks.

As the side chain length is increased, the honeycomb is replaced by new mesophases. The mesophase in compound **1 b,** having a slightly longer semiperfluorinated chain, grows with a completely dark texture under crossed polarizers (Figure S2b). This isotropic mesophase has high viscosity, which is typical of a cubic phase. The reciprocal *d*‐spacings from the SAXS reflections are in the ratio 2^1/2^:6^1/2^:8^1/2^: 14^1/2^:22^1/2^:26^1/2^:30^1/2^:34^1/2^:36^1/2^:38^1/2^:42^1/2^ and could be indexed on a body‐centered cubic lattice (for Miller indices see Figure 2 b). Alternative indexing on a primitive Bravais lattice, with the first reflection as (100) instead of (110), would have had the *a/d* of the 4^th^ reflection equal to 7^1/2^ instead of 14^1/2^, a (*h* ^2^+*k* ^2^+*l* ^2^)^1/2^ value unobtainable for any combination of Miller indices. Thus, a primitive lattice is excluded. Furthermore, due to the clear absence of the (200) reflection (*a*/*d=*4^1/2^), only one space group, *I*4_1_32, satisfies the observed extinction conditions, which include the 4_1_ screw axis condition 00*l*: *l*=4*n*. Note that the 9th peak can be indexed as either (600) or (442), hence its presence in powder SAXS does not violate the screw‐axis condition. Just to safeguard ourselves against the remote possibility that the absence of (200) is coincidental, we constructed the electron density map using *Im* $\bar{3}m$ symmetry. However, in this case we obtain a body centered micellar structure with the fluorinated chains forming the micelles (Figure S11a); the high curvature of the micelles is unlikely considering the molecular structure, volume fractions and dimensions. Accordingly, the mesophase in compound **1 b** is assigned as a bicontinuous cubic with symmetry *I*4_1_32 (Figures 2 d–g and S11b). Thus, we have the first documented case of a *I*4_1_32 cubic phase in liquid crystals, thermo‐ or lyotropic.

Since the intensities of all remaining reflections are smaller than 1 % of the strongest (110) reflection (Tables S2, S4 and S5), the electron density map is dominated by that reflection, whose phase is either +π/2 or −π/2 rad. The map constructed using one of these phases is simply the reverse of that obtained using the other, meaning that the maps are identical except for a change of origin. Different representations of the map are shown in Figure 2 d–g, where purple color indicates the regions of high, and red the regions of low electron density. The cyan intermediate density region follows the familiar gyroid surface of minimum curvature. The map is closely related to those of the double gyroid phase, except that in the latter case the color of the regions on both sides of the minimum surface would be the same, as both networks in the $Ia\bar{3}d$ phase have either lower or higher density than the minimal surface, depending on the compound. Thus, we can conclude that, as in the double gyroid, in our *I*4_1_32 phase there are two infinite networks with 3‐way branched channels separated by the gyroid surface. Only here one network contains the high‐density R_F_ and the other the low‐density R_H_ chains (Figure 2 d,f, respectively). In fact, the high‐density network is of a core–shell type, with the perfluorinated chain ends in the center of the channels surrounded by the short aliphatic spacers. The gyroid surface is composed of the glycerol‐terminated cores lying within it (Figure 2 e,g).

The distance between the two 3D networks is *a*_cub_×3^1/2^/4=4.0 nm, which is the same as the distance between the columns (the prismatic honeycomb cells) of the hexagonal phase of compound **1 a**, which is *a*_hex_/3^1/2^=4.0 nm (Figures S10 and S12). This equivalence is to be expected, as in both cases we have an “inverse” thermotropic LC phase in which columns of flexible chains are surrounded by “walls” of rigid aromatic‐glycerol rods; in the columnar case these are the honeycomb cell walls and in the cubic case the wall is the minimal surface of the same constitution.

To confirm our structure assignment in real space, compound **3** was imaged at temperatures of the cubic phase by atomic force microscopy (AFM). Images of two different crystallographic planes, (110) and (111), are shown in Figure 3 a,c. Phase contrast is due to the difference in shear modulus between the stiffer R_F_ chains (dark) and the softer R_H_ chains (light). A comparison with the corresponding cuts through the electron density map (Figure 3 b,d) confirms the general correspondence in geometry of the ED slices and the AFM images, the two methods agreeing in spacings and angles within 3 %. The measured distance between the rows of motifs in the (111) plane is 11.2 nm (Figure 3 c) while the value calculated from SAXS is $a\sqrt{6}/2=$ 10.9 nm, with the rows inclined by exactly 60°; the measured distance between the rows in the (110) plane of 7.0 nm (Figure 3 a) and the angle of 75° also compares well with the values of 6.8 nm and 73° measured from the (110) section through the map in Figures 3 b and S14. However, while the position of the dark R_F_ spots on the triangular lattice in Figure 3 c matches well with those of the centers of the 3‐arm stars in Figure 3 d, the star‐like feature is absent in the AFM image. We attribute this discrepancy to surface reconstruction, as broken network segments at the surface coalesce in blobs (white domes in Figure 3 e) to minimize their surface energy.

**Figure 3:**
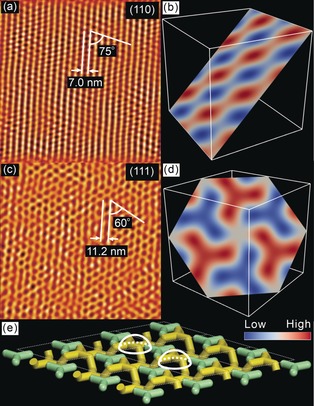
AFM phase images of the *I*4_1_32 phase of compound **3** recorded at 40 °C after cooling in situ from 110 °C (Iso) to 90 °C at 0.5 K min^−1^ and then at 3 K min^−1^ to 40 °C. a) a (110) plane, c) a (111) plane (Fourier filtered). b and d) show (110) and (111) cuts, respectively, through an electron density map (the 2×2×2=8 unit cell box). See also frontal view of b) with marked dimensions in Figure S14. e) Model of the surface layer of unit cells showing network segments as rods. The white domes are the suggested blobs of aggregated R_F_ chains seen as dark dots in (c).

Having established the structure of the new cubic LC, it is important to note that *I*4_1_32 is a chiral space group, and the phase is therefore chiral, even though the compounds forming it are achiral. The chirality comes from the fact that the two enantiomorphous networks are chemically different (see Figure 2 h, i) which breaks the mirror symmetry. In contrast, the achiral double gyroid $Ia\bar{3}d$ phase, having two identical but enantiomorphous networks, possesses a number of glide planes. This is the first case of chirality induction in LCs being exclusively mediated by nano‐phase separation8b, 14 of two chemically different molecular segments (the alkyl and perfluoroalky chains) into distinct nano‐scale domains (the two networks).

As in other cases of spontaneous mirror symmetry breaking, chirality is likely to be confined to individual cubic domains and, in the absence of an external chiral bias, is likely to be a conglomerate. However, as in the previous case of the chiral triple‐network cubic phase of achiral compounds, one chirality tends to win, eventually spreading over the entire sample in a variant of Ostwald ripening.^6^, ^15^

In contrast to the previously reported cases of cubic and other optically isotropic LC phases (refs. 5, 6, 15) optical activity or conglomerate formation cannot be observed by chiroptical methods in the *I*4_1_32 cubic phase reported here. The reason is that the chromophore is located on or close to the gyroid minimal surface, which is achiral, whereas the two chiral networks are filled with the alkyl chains and the fluorinated chains, respectively, whose absorption is far away from the spectral range investigated either by polarizing microscopy or by circular dichroism (CD). Hence, optical rotation and CD are negligible, leading to optical cryptochirality.^16^, ^17^ However optical activity and CD are only consequences of the lack of mirror symmetry and are not inevitable, whereas XRD provides a direct proof of chirality, and works irrespective of whether there is only one domain or a conglomerate. Because very strong peaks were observed, that are forbidden in the achiral $Ia\bar{3}d$ lattice, there is no doubt that mirror symmetry is broken.

Further extension of perfluorinated side chains removes the cubic phase and replaces it with a monotropic soft crystal phase in compound **1 c** having the largest total side chain volume. This phase appears on cooling and its SAXS Bragg reflections indicate a layer structure with a *d*=6.32 nm layer thickness. A single sharp peak in the wide‐angle region at *d*=0.51 nm (Figure S5) suggests a hexagonal arrangement of the perfluorinated chains lying perpendicular to the layers. Thus, compound **1 c** exhibits a soft lamellar crystal phase with ABCB stacking of four sublayers, where A=disordered aliphatic layer, B=disordered aromatic‐glycerol layer and C=crystallized fluorinated layer (Figures 4 i and S13). The fact that the alkyls are molten while the fluoroalkyls are not can be understood by comparing the melting points of polyethylene and Teflon of ca. 130 °C vs. ca. 300 °C, respectively. In fact, between 30 °C and 300 °C Teflon forms a hexagonal columnar phase^18^ with hexagonally arranged chains with irregular helix reversals.^19^

**Figure 4:**
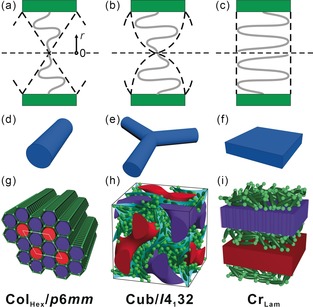
Phase sequence of polyphilic X‐shaped molecules with increasing side chain volume from left to right: a, d, g) hexagonal columnar honeycomb; b, e, h) alternating network gyroid cubic phase; c, f, i) lamellar phase. a) schematic of two of the six sectors of a hexagonal honeycomb cell; b) average cross‐section of a cubic network channel close to a horizontal junction; c) part of a (aliphatic) layer of the lamellar phase. d, e, f) A column, a network junction and a layer compared. g, h, i) Perceived molecular arrangement in the three phases: green=terphenyl cores; light green=glycerol groups; purple=semiperfluorinated side chains; red=alkyl chains (side‐chain regions are shown as continua).

The phase sequence Col_hex_–Cub_bi_–Lam with increasing side‐chain volume is consistent with the established effect of molecular geometry, once it is accepted that the present mesophases are of the “inverse” thermotropic kind. The present molecules could be regarded as having a tapered shape, but with the chain ends located on average near the narrow end and the rigid rod spanning the wide end of the wedge—see Figure 4 a–c. This is the opposite of the usual case of wedge‐shaped mesogens, where the aromatic is at the apex and the multiple attached chains fan out at the wide end.^20^ For such “normal” wedges it has been shown that a key determinant of the adopted phase type is the d*V*/d*r* function, describing how volume increases as one moves along *r* from the apex (*r*=0) toward the wide end of the wedge.^21^ The same principle can be applied to the present inverse wedges. As a first approximation, the cross‐section area *A* of the wedge is *A*(*r*) ∝ d*V*/d*r* ∝ *r* ^*q*^. Here *q*≈1 (slice or triangle shape) suites a columnar phase (Figure 4 a), *q*≈0 (rectangle, matching aromatic and aliphatic cross‐sections) suits a lamellar phase (Figure 4 c), while 0<*q*<1 (shield shape) is most likely to adopt a Cub_bi_ phase (Figure 4 b).^22^ It is worth noting that a network of branched columns in a Cub_bi_ phase is an intermediate between straight columns and layers, as depicted in Figure 4 d,e. A more detailed schematic of the molecular arrangement in the three phase types in the present compounds is given in Figure 4 g–i.

In conclusion, we present the first *I*4_1_32 alternating double network gyroid cubic LC. The work demonstrates a novel way of inducing chirality through phase separation in chemically non‐chiral systems. The network segments reported here are on the scale of only a few nm between junctions, which is of great potential in nano‐templating,12d for example as enantiomer‐specific membranes8c for use in enantiomer separation and production and manipulation of circularly polarized light, chirality switching through thermally, chemically or light‐induced mesophase transitions.^23^ Moreover, the present study shows how the principle of self‐assembled multicolor tiling can be extended from two dimensions (columnar) to three dimensions (cubic),^13^, ^24^ providing a new way to fabricate complex nano‐architectures.

## Conflict of interest

The authors declare no conflict of interest.

## Supporting information

As a service to our authors and readers, this journal provides supporting information supplied by the authors. Such materials are peer reviewed and may be re‐organized for online delivery, but are not copy‐edited or typeset. Technical support issues arising from supporting information (other than missing files) should be addressed to the authors.

Supplementary
