# Supplementary material for: Chirality Induction through Nano‐Phase Separation: Alternating Network Gyroid Phase by Thermotropic Self‐Assembly of X‐Shaped Bolapolyphiles
Source: Angew Chem Int Ed Engl. 2020 Jan 7;59(7):2725–9. doi: 10.1002/anie.201911245 (PMC7027881; doi:10.1002/anie.201911245)
Supplement: Supplementary file 1 — Supplementary [file ANIE-59-2725-s001.pdf]

Angewandte Chemie
Eine Zeitschrift der Gesellschaft Deutscher Chemiker

Supporting Information

Chirality Induction through Nano-Phase Separation: Alternating Network Gyroid Phase by Thermotropic Self-Assembly of X-Shaped Bolapolyphiles

Changlong Chen, Robert Kieffer, Helgard Ebert, Marko Prehm, Rui-bin Zhang, Xiangbing Zeng, Feng Liu, Goran Ungar,* and Carsten Tschierske**

anie_201911245_sm_miscellaneous_information.pdf

1. Syntheses and analytical data of the materials

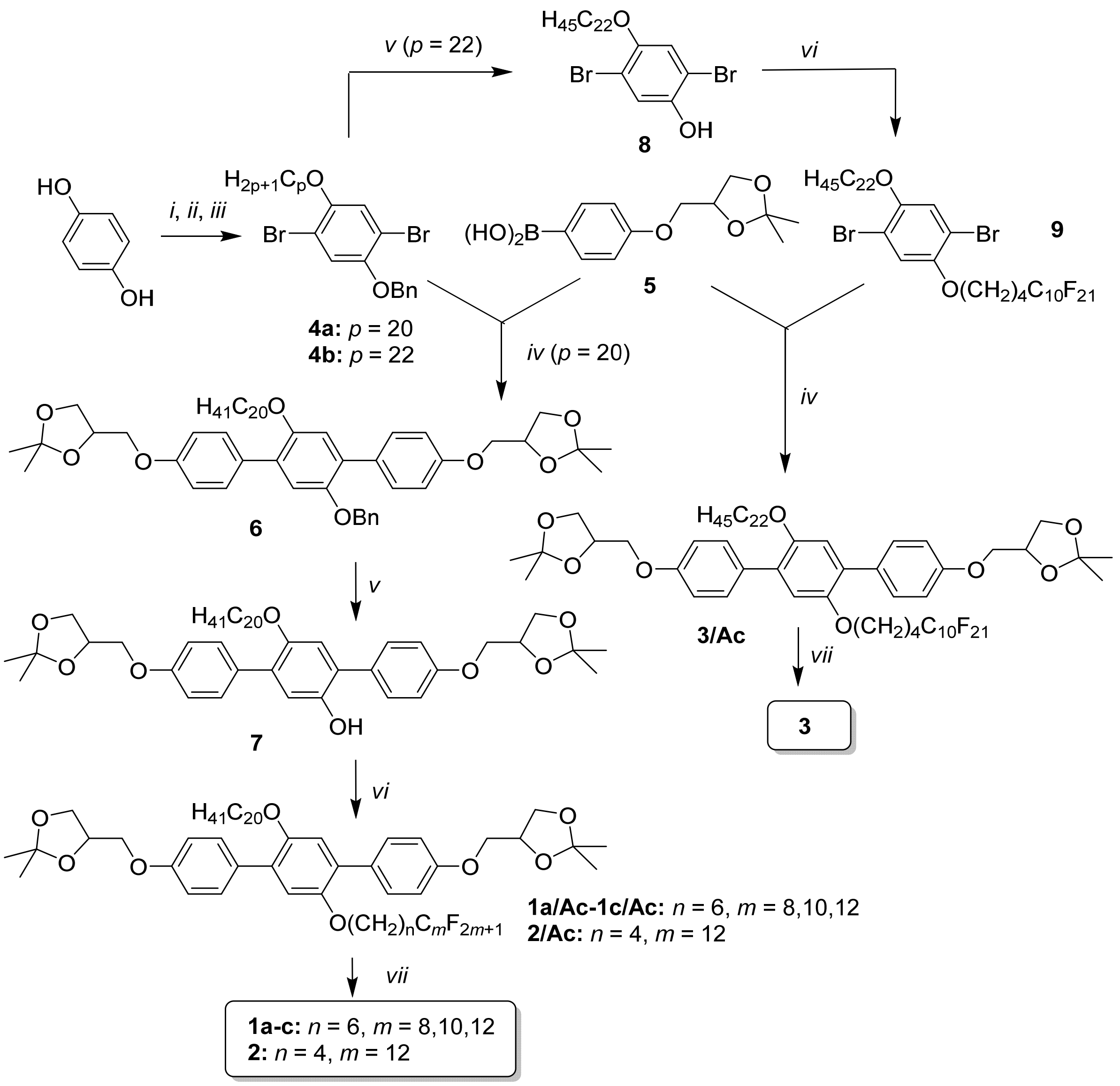

Scheme S1. Synthesis of compounds **1a-c**, **2** and **3**. Reagents and conditions: *i*) Br_2, AcOH, r.t., 1h; *ii*) BnCl, K_2CO_3, MeOH, reflux, 30 min; *iii*) $C_pH_{2p+1}Br$, K_2CO_3, DMF, 80 °C, 6 h; *iv*) $Pd(PPh_3)_4$, $NaHCO_3$, glyme, reflux, 10 h; *v*) H_2, Pd/C, EtOAc, 45 °C, 3.2 bar, 8 h; *vi*) $C_mF_{2m+1}(CH_2)_nBr$ (**C_6F_mBr**[S1]; **C_4F_{12}Br**[S2]), K_2CO_3, DMF, 80 °C, 6 h; *vii*) cat. HCl, MeOH, reflux, 6 h.

1.1 Intermediates

1.1.1 4-Benzyloxy-2,5-dibromophenol[S3]

To a solution of 2,5-dibromohydrochinone (22.4 g; 83.6 mmol) and K_2CO_3 (11.55 g; 83.6 mmol) in dry MeOH (150 ml) while stirring was added benzyl chloride (10.6 g; 83.6 mmol; dissolved in 30 ml of dry MeOH) through a dropping funnel under an argon atmosphere. The mixture was refluxed for 30 min. After cooling to room temperature the precipitated

dibenzylated by-product was filtered off and the filter cake was washed with a small amount of MeOH. The reaction mixture was neutralised by addition of diluted hydrochloric acid and the solvent was evaporated under reduced pressure. The residue was dissolved in ethyl acetate (200 ml) and washed with H_2O (1x), sat. aqu. $NaHCO_3$ solution (2x) and brine (1x) with 50 ml each. The organic phase was dried over Na_2SO_4, filtered and the solvent was removed under reduced pressure. The crude product was purified by crystallisation (2x) from $CHCl_3$/petrol ether = 5:1, v/v. Yield: 9.1 g (30 %), colorless solid; mp.: 111-112 °C[S2]; ^{1}H-NMR ($CDCl_3$, 400 MHz): δ 7.45-7.40 (m, 2H, Ar-H), 7.39-7.36 (m, 2H, Ar-H), 7.34-7.30 (m, 1H, Ar-H), 7.25 (s, 1H, Ar-H), 7.03 (s, 1H, Ar-H), 5.16 (s, 1H, OH), 5.04 ppm (s, 2H, CH_2).

1.1.2 4-Benzyloxy-2,5-dibromo-1-alkoxybenzenes (4)

4-Benzyloxy-2,5-dibromo-1-eicosyloxybenzene (4a). To a solution of 4-benzyloxy-2,5-dibromophenol (2.0 g; 5.6 mmol) in dry CH_3CN (50 ml) 1-bromoeicosane (2.22 g; 6.14 mmol) was added under an argon atmosphere. After the addition of K_2CO_3 (3.86 g; 28 mmol) and Bu_4NI (100 mg) the mixture was refluxed for 6 hours. After cooling to 20 °C H_2O (50-70 ml) was added and the mixture was stirred for 15 minutes. The precipitated product was filtered off and washed with a small amount of H_2O. The crude product was purified by crystallisation from petrol ether; Yield: 3.35 g (94 %), colorless solid; mp.: 73.5 °C; ^{1}H-NMR ($CDCl_3$, 400 MHz): δ = 7.45-7.43 (m, 2H, Ar-H), 7.39-7.35 (m, 2H, Ar-H), 7.33-7.31 (m, 1H, Ar-H), 7.14 (s, 1H, Ar-H), 7.09 (s, 1H, Ar-H), 5.05 (s, 2H, OC**H₂**Ph), 3.94 (t, ^3J = 6.43 Hz, 2H, OCH_2), 1.80-1.77 (m, 2H, OCH_2**CH_2**), 1.48-1.44 (m, 2H, OCH_2CH_2**CH_2**), 1.30-1.24 (m, 32H, CH_2), 0.86 ppm (t, ^3J = 6.85 Hz, 3H, CH_3).

4-Benzyloxy-2,5-dibromo-1-docosyloxybenzene (4b). To a solution of 4-benzyloxy-2,5-dibromophenol (2.0 g; 5.6 mmol) in dry CH_3CN (50 ml) 1-bromodocosane (2.39 g; 6.14 mmol) was added under an argon atmosphere. After the addition of K_2CO_3 (3.86 g; 28 mmol) and Bu_4NI (100 mg) the mixture was refluxed for 6 hours. After cooling to 20 °C H_2O (50-70 ml) was added and the mixture was stirred for 15 minutes. The precipitated product was filtered off and washed with a small amount of H_2O. The crude product was purified by crystallisation from petrol ether; Yield: 3.22 g (86 %), colorless solid; mp.: 70-72 °C; ^{1}H-NMR ($CDCl_3$, 400 MHz): δ = 7.48-7.27 (m, 5H, Ar-H), 7.14 (s, 1H, Ar-H), 7.08 (s, 1H, Ar-H), 5.05 (s, 2H, OC**H₂**Ph), 3.93 (t, ^3J = 6.5 Hz, 2H, OCH_2), 1.85-1.72 (m, 2H, OCH_2**CH_2**), 1.51-1.39 (m, 2H, OCH_2CH_2**CH_2**), 1.37-1.16 (m, 36H, CH_2), 0.86 ppm (t, ^3J = 6.8 Hz, 3H, CH_3).

1.1.3 4,4′′-Bis(2,2-dimethyl-1,3-dioxolane-4-ylmethoxy)-5’-eicosyloxy-*p*-terphenyl-2’-ol (7)

5’-Benzyloxy-4,4′′-bis(2,2-dimethyl-1,3-dioxolane-4-ylmethoxy)-2’-eicosyloxy – *p* -terphenyl (6). Under an argon atmosphere 4a (3.0 g; 4.7 mmol) and 5[S4]; (2.61 g; 10.34 mmol) were dissolved in ethyleneglycoldimethylether (100 ml). The same volume of sat. aqu. $NaHCO_3$ solution was added under an argon atmosphere. After addition of the catalyst $Pd(PPh_3)_4$ (165 mg; 0.14 mmol) the reaction mixture was refluxed for 10 h. After cooling to room temperature, the solvent was evaporated and the residue was dissolved in chloroform. The resulting solution was washed with H_2O and brine. After separation and drying over Na_2SO_4 the solvent was evaporated. The crude product was purified by column chromatography with $CHCl_3$ as solvent and finally crystallised from petrol ether. Yield: 3.5 g (83 %), colorless solid; mp.: 88.5 °C; ^{1}H-NMR ($CDCl_3$, 400 MHz): δ 7.54 (d, ^3J = 8.51 Hz, 2H, Ar-H), 7.47 (d, ^3J = 8.72 Hz, 2H, Ar-H), 7.30-

7.26 (m, 5H, Ar-H), 6.99 (s, 1H, Ar-H), 6.96-6.92 (m, 5H, Ar-H), 4.96 (s, 2H, Bn-CH_2), 4.51-4.48 (m, 2H, CHO), 4.19-4.15 (m, 2H, CH_2O), 4.11-4.08 (m, 2H, CH_2O), 3.99-3.95 (m, 2H, CH_2O), 3.93-3.87 (m, 4H, CH_2O), 1.70-1.63 (m, 2H, OCH_2CH_2), 1.47 (s, 6H, CH_3), 1.40 (s, 6H, CH_3), 1.35-1.24 (m, 34H, CH_2), 0.86 ppm (t, ^3J = 6.85 Hz, 3H, CH_3).

4,4´´-Bis(2,2-dimethyl-1,3-dioxolane-4-ylmethoxy)-5'-eicosyloxy-p-terphenyl-2'-ol (7). Under an argon atmosphere **6** (3.5 g; 3.9 mmol) was dissolved in ethyl acetate (100 ml) and Pd/C (200 mg, 10 % Pd) was added. After rinsing with hydrogen (3x) the hydrogen pressure was set to 3.2 bar and the temperature was set to 45 °C. After 8 h the solution was filtered and the filtrate was washed with hot ethyl acetate. The solvent was evaporated and the crude product was purified by crystallisation from ethyl acetate/petrol ether = 5:1, v/v. Yield: 3.1 g (99 %), colorless solid; mp.: 70 °C; ^{1}H-NMR ($CDCl_3$, 400 MHz): δ 7.50 (d, ^3J = 8.72 Hz, 2H, Ar-H), 7.42 (d, ^3J = 8.72 Hz, 2H, Ar-H), 7.02 (d, ^3J = 8.51 Hz, 2H, Ar-H), 6.92 (s, 1H, Ar-H, overlapped), 6.93 (d, ^3J = 8.72 Hz, 2H, Ar-H), 6.81 (s, 1H, Ar-H), 4.89 (s, 1H, OH), 4.52-4.46 (m, 2H, CHO), 4.19-4.15 (m, 2H, CH_2O), 4.12-4.07 (m, 2H, CH_2O), 4.00-3.94 (m, 2H, CH_2O), 3.93-3.89 (m, 2H, CH_2O), 3.84 (t, ^3J = 6.43 Hz, 2H, CH_2O), 1.69-1.62 (m, 2H, $OCH_2\mathbf{CH_2}$), 1.47 (s, 6H, CH_3), 1.40 (s, 6H, CH_3), 1.35-1.24 (m, 34H, CH_2), 0.86 ppm (t, ^3J = 6.85 Hz, 3H, CH_3).

1.1.4 2,5-Dibromo-4-docosyloxyphenol (8)

Under an argon atmosphere **4b** (2.0 g; 3.0 mmol) was dissolved in THF (100 ml) and Pd/C (0.3 g, 10 % Pd) was added. After rinsing with hydrogen (3x) the hydrogen pressure was set to 3.2 bar and the temperature was set to 45 °C. After 8 h the solution was filtered and the filtrate was washed with hot ethyl acetate. The solvent was evaporated and the crude product was purified by crystallisation from *n*-hexane. Yield: 0.51 g (29 %), colorless solid; mp.: 78-80 °C; ^{1}H NMR (400 MHz, $CDCl_3$) δ 7.22 (s, 1H, Ar-*H*), 6.96 (s, 1H, Ar-*H*), 5.09 (s, 1H, O*H*), 3.91 (t, *J* = 6.5 Hz, 2H, OCH_2), 1.87 – 1.68 (m, 2H, OCH_2CH_2), 1.50 – 1.40 (m, 2H, $OCH_2CH_2CH_2$), 1.39 – 1.20 (m, 36H, $OCH_2CH_2CH_2(CH_2)_{18}$), 0.86 (t, *J* = 6.8 Hz, 3H, CH_2CH_3).

1.1.5 Semiperfluorinated alkyl bromides C_nF_mBr

14-Bromo-1,1,1,2,2,3,3,4,4,5,5,6,6,7,7,8,8,9,9,10,10-henicosafluorotetradecane ($C_4F_{10}Br$)

5,5,6,6,7,7,8,8,9,9,10,10,11,11,12,12,13,13,14,14,14-Henicosafluorotetradecane-1-ol ($C_4F_{10}OH$). The Pd° catalyzed addition of perfluoroalkyliodides to the double bonds, followed by reduction, as reported by JOHANSSON et al.[S5] was used for the synthesis of the semiperfluorinated alcohol. Accordingly, under an argon atmosphere but-3-en-1-ol (0.89 g; 12.4 mmol) was dissolved in dry hexane (100 ml) and the solution was degassed in an ultra sonic bath under an argon atmosphere for 30 min. After the addition of 1,1,1,2,2,3,3,4,4,5,5,6,6,7,7,8,8,9,9,10,10-henicosafluoro-10-iododecane (8.0 g; 12.4 mmol) the mixture was degassed further for 5 min. Then the mixture was cooled to *T* = -100 °C and the flask was evaporated, refilled with argon and warmed up to room temperature. This procedure was repeated three times. Then the solution was allowed to come to *T* = 0 °C and the catalyst $Pd(PPh_3)_4$ (0.39 g; 0.62 mmol) was added under an argon atmosphere. The mixture was stirred at r.t. for 100 hours. After this the mixture was filtered through silica gel and the residue was washed with Et_2O (150 ml). The solvent was removed und reduced pressure and the residue was taken up in Et_2O (50 ml). This solution was added dropwise to a suspension of $LiAlH_4$ (0.47 g; 12.4 mmol) in dry Et_2O (50 ml) at such a rate to maintain the

solution at reflux. The mixture was heated to reflux for additionally 6 h, cooled to room temperature and the unreacted $LiAlH_4$ was hydrolyzed by careful addition of water. Then 30 % aqueous H_2SO_4 (20 ml) was added to dissolve all precipitated solids. The organic layer was separated and the aqueous layer was extracted with Et_2O (3x50 ml), the combined organic layers were washed with 10 % aqueous $Na_2S_2O_3$ until the aqueous layer remained colorless. After washing with water (2x100 ml) and brine (100 ml), the solution was dried over anhydrous Na_2SO_4, the solvent was removed and the crude product was purified by column chromatography (silica gel, eluent: $CHCl_3$/MeOH = 10/0.2 (v/v). Yield: 5.63 g (77 %), colorless solid; mp.: 67-68 °C; ^{1}H-NMR ($CDCl_3$, 400 MHz): δ = 3.62 (t, ^3J = 6.60 Hz, 2H, C**H₂**OH), 2.10-1.98 (m, 2H, CH_2CF_2), 1.75-1.60 (m, 4H, **CH_2**CH_2OH, **CH_2**CH_2CF_2).

14-Bromo-1,1,1,2,2,3,3,4,4,5,5,6,6,7,7,8,8,9,9,10,10-henicosafluorotetradecane ($C_4F_{10}Br$). To a suspension of **$C_4F_{10}OH$** (5.6 g; 9.50 mmol) in HBr (47 %; 25 ml) Bu_4NHSO_4 (0.25 g) and conc. H_2SO_4 (5 ml) was added. The mixture was stirred at T = 100 °C for 24 hours and then cooled to room temperature. After addition of water (50 ml) and extraction with Et_2O (3x 20 ml) the combined organic layers were washed with water (50 ml) and dried over anhydrous Na_2SO_4. The solvent was removed under reduced pressure and the crude product was purified by sublimation at 80 – 100 °C under vacuum. Yield: 3.7 g (59 %), colorless solid; mp.: 66-69 °C; ^{1}H-NMR ($CDCl_3$, 400 MHz): δ = 3.41 (t, ^3J = 6.50 Hz, 2H, CH_2OH), 2.09 (m, 2H, CH_2CF_2), 1.95 (quint, ^3J = 7.10 Hz, 2H, **CH_2**CH_2OH), 1.78 (m, 2H, **CH_2**CH_2CF_2).

$C_4F_{12}Br$ and compounds **C_6F_mBr** were synthesized in an analogous way as described previously.[S1,S2]

1.1.6 1,4-Dibromo-5-docosyloxy-2-(5,5,6,6,7,7,8,8,9,9,10,10,11,11,12,12,13, 13,14,14,14-henicosafluortetradecyloxy)benzene (9)

Synthesized and purified according to the procedure described for **4a** from **8** (0.24 g; 0.42 mmol), **$C_4F_{10}Br$** (0.27 g; 0.42 mmol), K_2CO_3 (0.29 g; 2.1 mmol), Bu_4NI (100 mg) in CH_3CN (50 ml). Yield: 1.77 mg (37 %), colorless solid; mp.: 93-95 °C; ^{1}H-NMR ($CDCl_3$, 400 MHz): ^{1}H NMR (400 MHz, $CDCl_3$) δ 7.07 (s, 2H, Ar-H), 3.98 (t, J = 5.7 Hz, 2H, OCH_2), 3.93 (t, J = 6.6 Hz, 2H, OCH_2), 2.29 – 2.09 (m, 2H, CH_2CF_2), 1.94 – 1.82 (m, 4H, OCH_2C**H_2**), 1.83 – 1.73 (m, 2H **CH_2**CH_2CF_2), 1.51 – 1.40 (m, 2H, OCH_2CH_2C**H_2**), 1.38 – 1.08 (m, 36H, $OCH_2CH_2CH_2(C$**H_2**$)_{18}$), 0.86 (t, J = 6.8 Hz, 3H, CH_3).

1.2 4,4´´-Bis(2,2-dimethyl-1,3-dioxolane-4-ylmethoxy)-2'-semiperfluoroalkoxy)-5'-alkyloxy-*p*-terphenyls

4,4´´-Bis(2,2-dimethyl-1,3-dioxolane-4-ylmethoxy)-5'-eicosyloxy-2'-(7,7,8,8,9,9,10,10,11,-11,12,12,13,13,14,14,14-heptadecafluorotetradecyloxy)-*p*-terphenyl (1a/Ac)

Under an argon atmosphere **7** (250 mg; 0.31 mmol) was dissolved in dry DMF (50 ml). After the addition of **C_6F_8Br** (191 mg; 0.33 mmol) K_2CO_3 (430 mg; 3.1 mmol) and Bu_4NI (50 mg), the reaction mixture was stirred at T = 80 °C for 6 hours. After cooling to room temperature H_2O (150 ml) was added and the precipitated product was filtered of and washed three times with water. The solid was dissolved in $CHCl_3$, filtered and the solvent was removed in vacuo. The crude product was purified by preparative centrifugal thin layer chromatography (Chromatotron) on silica gel (silica gel 60 (PF254, *Merck*) with petrol ether/$CHCl_3$ = 1/1-0/1 (V/V) as eluent. Yield: 376 mg (93 %), colorless solid; mp.: 92-93 °C; ^{1}H-NMR ($CDCl_3$, 500 MHz): δ = 7.52-7.48 (m, 4H, Ar-H), 6.94 (d, ^3J = 8.70 Hz, 4H, Ar-H), 6.91 (s, 2H, Ar-H),

4.51-4.47 (m, 2H, CHO), 4.19-4.15 (m, 2H, CH_2O), 4.11-4.07 (m, 2H, CH_2O), 3.98-3.86 (m, 8H, CH_2O), 2.05-1.93 (m, 2H, CH_2CF_2), 1.68-1.63 (m, 4H, OCH_2**CH_2**), 1.57-1.53 (m, 2H, **CH_2**CH_2CF_2), 1.47 (s, 3H, CH_3), 1.46 (s, 3H, CH_3), 1.40 (s, 6H, CH_3), 1.38-1.24 (m, 38H, CH_2), 0.86 ppm (t, ^3J = 6.94 Hz, 3H, CH_3); ^{19}F-NMR ($CDCl_3$, 470 MHz): δ = -81.18 (t, ^3J = 9.22 Hz, 3F, CF_3), -114.68 (s, 2F, CH_2CF_2), -122.23 (s, 6F, CF_2), -123.08 (s, 2F, CF_2), -123.86 (s, 2F, CF_2), -126.46 ppm (s, 2F, **CF_2**CF_3).

4,4′′-Bis(2,2-dimethyl-1,3-dioxolane-4-ylmethoxy)-5'-eicosyloxy-2'-(7,7,8,8,9,9,10,10,11,-11,12,12,13,13,14,14,15,15,16,16,16-henicosafluorohexadecyloxy)-*p*-terphenyl (1b/Ac)

Synthesized and purified according to the procedure described above from **7** (250 mg; 0.31 mmol), **$C_6F_{10}Br$** (223 mg; 0.33 mmol), K_2CO_3 (430 mg; 3.1 mmol), Bu_4NI (50 mg), DMF (50 ml). Yield: 400 mg (91 %), colorless solid; mp.: 98-100 °C; ^{1}H-NMR ($CDCl_3$, 400 MHz): δ = 7.51-7.48 (m, 4H, Ar-H), 6.94 (d, ^3J = 8.51 Hz, 4H, Ar-H), 6.91 (s, 2H, Ar-H), 4.51-4.47 (m, 2H, CHO), 4.19-4.15 (m, 2H, CH_2O), 4.12-4.07 (m, 2H, CH_2O), 3.99-3.85 (m, 8H, CH_2O), 2.03-1.97 (m, 2H, CH_2CF_2), 1.67-1.63 (m, 4H, OCH_2**CH_2**), 1.58-1.52 (m, 2H, **CH_2**CH_2CF_2), 1.47 (s, 3H, CH_3), 1.46 (s, 3H, CH_3), 1.40 (s, 6H, CH_3), 1.38-1.24 (m, 38H, CH_2), 0.86 ppm (t, ^3J = 6.85 Hz, 3H, CH_3); ^{19}F-NMR ($CDCl_3$, 470 MHz): δ = -81.17 (t, ^3J = 9.91 Hz, 3F, CF_3), -114.69 (s, 2F, CH_2CF_2), -122.13 (s, 10F, CF_2), -123.05 (s, 2F, CF_2), -123.84 (s, 2F, CF_2), -126.48 ppm (s, 2F, **CF_2**CF_3).

4,4′′-Bis(2,2-dimethyl-1,3-dioxolane-4-ylmethoxy)-5'-eicosyloxy-2'-(7,7,8,8,9,9,10,10,11,-11,12,12,13,13,14,14,15,15,16,16,17,17,18,18,18-pentacosafluorooctadecyloxy)-*p*-terphenyl (1c/Ac)

Synthesized and purified according to the procedure described above from **7** (200 mg; 0.25 mmol), **$C_6F_{12}Br$** (205 mg; 0.26 mmol), K_2CO_3 (345 mg; 2.5 mmol), Bu_4NI (50 mg), DMF (50 ml). Yield: 288 mg (77 %), colorless solid; mp.: 109 °C; ^{1}H-NMR ($CDCl_3$, 400 MHz): δ = 7.52-7.48 (m, 4H, Ar-H), 6.94 (d, ^3J = 8.72 Hz, 4H, Ar-H), 6.91 (s, 2H, Ar-H), 4.51-4.46 (m, 2H, CHO), 4.19-4.15 (m, 2H, CH_2O), 4.12-4.07 (m, 2H, CH_2O), 3.99-3.85 (m, 8H, CH_2O), 2.06-1.93 (m, 2H, CH_2CF_2), 1.69-1.62 (m, 4H, OCH_2**CH_2**), 1.56-1.51 (m, 2H, **CH_2**CH_2CF_2), 1.47 (s, 3H, CH_3), 1.46 (s, 3H, CH_3), 1.40 (s, 6H, CH_3), 1.36-1.24 (m, 38H, CH_2), 0.86 ppm (t, ^3J = 6.85 Hz, 3H, CH_3); ^{19}F-NMR ($CDCl_3$, 188 MHz): δ = -81.18 (t, ^3J = 9.91 Hz, 3F, CF_3), -114.71 (s, 2F, CH_2CF_2), -122.08 (s, 14F, CF_2), -123.05 (s, 2F, CF_2), -123.86 (s, 2F, CF_2), -126.46 ppm (s, 2F, **CF_2**CF_3).

4,4′′-Bis(2,2-dimethyl-1,3-dioxolane-4-ylmethoxy)-5'-eicosyloxy-2'-(5,5,6,6,7,7,8,8,9,9,10,10,11,11,12,12,13,13,14,14,15,15,16,16,16-pentacosafluorohexadecyloxy)-*p*-terphenyl (2/Ac)

: Synthesized and purified according to the procedure described above from **7** (200 mg; 0.25 mmol), **$C_4F_{12}Br$** (204 mg; 0.26 mmol), K_2CO_3 (346 mg; 2.5 mmol), Bu_4NI (50 mg), CH_3CN (50 ml). Yield: 193 mg (52 %), colorless solid; mp.: 114-116 °C; ^{1}H-NMR ($CDCl_3$, 400 MHz): δ ^{1}H NMR (400 MHz, $CDCl_3$) δ 7.51 – 7.45 (m, 4H, Ar-H), 6.95 – 6.92 (m, 4H, Ar-H), 6.91 (s, 2H, Ar-H), 4.52 – 4.44 (m, 2H, CH), 4.20 – 4.13 (m, 2H, OCH_2), 4.13 – 4.07 (m, 2H, CH_2O), 4.00 – 3.87 (m, 8H, CH_2O, $OC\mathit{H_2}$, O**CH_2**CH_2), 2.11 – 1.95 (m, 2H, C**H_2**CF_2), 1.78 – 1.62 (m, 6H, C**H_2**CH_2CF_2, OCH_2C**H_2**), 1.46 (d, J = 3.7 Hz, 6H, C$\mathit{H_3}$), 1.40 (d, J = 1.8 Hz, 6H, CH_3), 1.39 – 1.19 (m, 34H, OCH_2CH_2(C**H_2**)$_{17}$), 0.86 (t, J = 7.0 Hz, 3H, CH_2C**H_3**).

4,4''-Bis(2,2-dimethyl-1,3-dioxolan-4-ylmethoxy)-5'-docosyloxy-2'-(5,5,6,6,7,7,8,8,9,9,10,10,11,11,12,12,13,13,14,14,14-henicosafluortetradecyloxy)-p terphenyl (3/Ac)

Under an argon atmosphere **9** (160 mg; 0.14 mmol) and **5**[S4]; (77 mg; 0.31 mmol) were dissolved in THF (30 ml). The same volume of sat. aqu. $NaHCO_3$ solution was added under an argon atmosphere. After addition of the catalyst $Pd(PPh_3)_4$ (5 mg) the reaction mixture was refluxed for 10 h. After cooling to room temperature, the solvent was evaporated and the residue was dissolved in chloroform. The resulting solution was washed with H_2O and brine. After separation and drying over Na_2SO_4 the solvent was evaporated. The crude product was purified by column chromatography with *n*-hexane/MeOH = 2/1 (vv) as solvent and finally crystallised from $CHCl_3$/MeOH. Yield: 115 mg (58 %), colorless solid; mp.: 94-98 °C; ^{1}H-NMR ($CDCl_3$, 400 MHz): δ 7.53 – 7.44 (m, 4H, Ar-H), 6.98 – 6.92 (m, 4H, Ar-H), 6.91 (s, 2H, Ar-H), 4.54 – 4.44 (m, 2H, CH), 4.22 – 4.13 (m, 2H, OCH_2), 4.13 – 4.04 (m, 2H, CH_2O), 4.00 – 3.84 (m, 8H, CH_2O, OCH_2, $O\mathbf{CH_2}CH_2$), 2.10 – 1.93 (m, 2H, $\mathbf{CH_2}CF_2$), 1.78 – 1.61 (m, 6H, $\mathbf{CH_2}CH_2CF_2$, $OCH_2\mathit{CH_2}$), 1.46 (d, *J* = 3.5 Hz, 6H, $\mathit{CH_3}$), 1.40 (d, *J* = 1.5 Hz, 6H, CH_3), 1.37 – 1.16 (m, 38H, $OCH_2CH_2(\mathbf{CH_2})_{19}$), 0.86 (t, *J* = 6.8 Hz, 3H, $CH_2\mathbf{CH_3}$).

1.3 Compounds 1a-c, 2 and 3

To a solution of the appropriate bisacetonide (**1/Ac - 3/Ac**) in MeOH (50 ml) 10% HCl (10 ml) was added and the mixture was refluxed for 6 h. After cooling to room temperature sat. aqu. $NaHCO_3$ was added and the solvent removed in vacuo. The resulting suspension was filtered and the product was washed several times with water (30 ml each) and dried. The crude product was purified by preparative centrifugal thin layer chromatography (eluents: $CHCl_3$/MeOH = 10/0.3, v/v) or crystallisation from MeOH.

3-[4′′-(2,3-Dihydroxypropoxy)-5'-eicosyloxy-2'-(7,7,8,8,9,9,10,10,11,11,12,12,13,13,-14, 14,14-heptadecafluorotetradecyloxy)-p-terphenyl-4-yloxy]propane-1,2-diol (1a)

Synthesized from **1a/Ac** (370 mg; 0.28 mmol); purified by crystallisation from MeOH. Yield: 285 mg (82 %), colorless solid; for phase transitions see Tab. 1. ^{1}H-NMR ($CDCl_3$, 400 MHz): δ = 7.51 (d, ^3J = 8.61 Hz, 4H, Ar-H), 6.94 (d, ^3J = 8.72 Hz, 4H, Ar-H), 6.91 (s, 2H, Ar-H), 4.13-4.10 (m, 2H, CHO), 4.09-4.04 (m, 4H, CH_2OH), 3.88-3.83 (m, 6H, CH_2O), 3.78-3.74 (m, 2H, CH_2O), 2.35 (bs, 4H, OH), 2.06-1.93 (m, 2H, CH_2CF_2), 1.69-1.62 (m, 4H, $OCH_2\mathbf{CH_2}$), 1.58-1.51 (m, 2H, $\mathbf{CH_2}CH_2CF_2$), 1.36-1.24 (m, 38H, CH_2), 0.86 ppm (t, ^3J = 6.74 Hz, 3H, CH_3); ^{13}C-NMR ($CDCl_3$, 125 MHz): δ = 157.51, 157.47 (C-4,21), 150.35, 150.07 (C-11,14), 131.51, 131.49 (C-9,16), 130.65 (C-7,8,17,18), 130.0, 129.91 (C-10,15), 116.22, 116.03 (C-5,6,19,20), 114.02, 113.99 (C-12,13), 70.43 (C-2,23), 69.55, 69.38 (C-3,22), 69.25, 69.23 (CH_2O), 63.69, 63.65 (C-1,24), 31.90, 30.79, 29.70, 29.68, 29.64, 29.60, 29.34, 29.29, 29.10, 28.73, 26.07, 25.79, 22.67, 20.07 (CH_2), 14.07 ppm (CH_3); ^{19}F-NMR ($CDCl_3$, 188 MHz): δ = -81.20 (t, ^3J = 9.25 Hz, 3F, CF_3), -114.70 (s, 2F, CH_2CF_2), -122.26 (s, 6F, CF_2), -123.10 (s, 2F, CF_2), -123.87 (s, 2F, CF_2) -126.49 ppm (s, 2F, $\mathbf{CF_2}CF_3$); calculated for $C_{55}H_{77}F_{17}O_8$: C 56.86, H 6.33; found: C 56.81, H 6.43 %.

3-[4′′-(2,3-Dihydroxypropoxy)-5'-eicosyloxy-2'-(7,7,8,8,9,9,10,10,11,11,12,12,13,13,14,14,15,15,16,16,16-henicosafluorohexadecyloxy)-*p*-terphenyl-4-yloxy]propane-1,2-diol (1b)

Synthesized from **1b/Ac** (395 mg; 0.28 mmol); purified by preparative centrifugal thin layer chromatography. Yield: 320 mg (86 %), colorless solid; for phase transitions see Tab. 1. ^{1}H-

NMR (acetone-d_6, 400 MHz): δ = 7.55 (d, ^3J = 8.82 Hz, 4H, Ar-H), 7.00 (s, 2H, Ar-H), 6.98 (d, ^3J = 8.92 Hz, 4H, Ar-H), 4.13-4.10 (m, 2H, CHO), 4.06-3.94 (m, 10H, CH_2OH, CH_2O), 3.73-3.67 (m, 6H, CH_2O, OH), 2.26-2.12 (m, 2H, CH_2CF_2), 1.73-1.65 (m, 4H, $OCH_2\mathbf{CH_2}$), 1.62-1.58 (m, 2H, $\mathbf{CH_2}CH_2CF_2$), 1.47-1.38 (m, 6H, CH_2), 1.28-1.23 (m, 32H, CH_2), 0.87 ppm (t, ^3J = 6.85 Hz, 3H, CH_3); ^{13}C-NMR (acetone-d_6, 125 MHz): δ = 159.13, 159.11 (C-4,21), 151.19, 151.10 (C-11,14), 131.80 (C-9,16), 131.36 (C-7,8,17,18), 130.69 (C-10,15), 119.83, 116.73, 116.67 (C-5,6,19,20), 114.77 (C-12,13), 71.41 (C-2,23), 70.37, 70.35 (C-3,22), 69.95, 69.84 (CH_2O), 64.18, 64.16 (C-1,24), 32.61, 31.36, 31.19, 31.01, 30.34, 30.32, 30.29, 30.26, 30.11, 30.05, 29.95, 29.33, 26.83, 26.51, 23.30, 20.86 (CH_2), 14.32 ppm (CH_3); ^{19}F-NMR (acetone-d_6, 188 MHz): δ = -82.05 (t, ^3J = 9.91 Hz, 3F, CF_3), -115.04 (s, 2F, CH_2CF_2), -122.63 (s, 10F, CF_2), -123.59 (s, 2F, CF_2), -124.33 (s, 2F, CF_2) -127.08 ppm (s, 2F, $\mathbf{CF_2}CF_3$); calculated for $C_{60}H_{77}F_{21}O_8$: C 54.38, H 5.86; found: C 54.08, H 6.29 %.

3-[4´´-(2,3-Dihydroxypropoxy)-5'-eicosyloxy-2'-(7,7,8,8,9,9,10,10,11,11,12,12,13,13,14,14,15,15,16,16,17,17,18,18,18-pentacosafluorooctadecyloxy)-*p*-terphenyl-4-yloxy]-propane-1,2-diol (1c)

Synthesized from **1c/Ac** (285 mg; 0.19 mmol); purified by crystallisation from MeOH. Yield: 230 mg (85 %), colorless solid; for phase transitions see Tab. 1. ^{1}H-NMR (acetone-d_6, 400 Mhz): δ = 7.54 (d, ^3J = 8.83 Hz, 4H, Ar-H), 6.99 (s, 2H, Ar-H), 6.98 (d, ^3J = 8.92 Hz, 4H, Ar-H), 4.14-4.10 (m, 2H, CHO), 4.09-3.93 (m, 10H, CH_2OH, CH_2O), 3.75-3.62 (m, 6H, CH_2O, OH), 2.25-2.12 (m, 2H, CH_2CF_2), 1.74-1.64 (m, 4H, $OCH_2\mathbf{CH_2}$), 1.61-1.50 (m, 2H, $\mathbf{CH_2}CH_2CF_2$), 1.46-1.37 (m, 6H, CH_2), 1.36-1.22 (m, 32H, CH_2), 0.86 ppm (t, ^3J = 6.85 Hz, 3H, CH_3); ^{13}C-NMR (acetone-d_6, 100 MHz): δ = 159.02 (C-4,21), 151.16, 151.05 (C-11,14), 131.77 (C-9,16), 131.23 (C-7,8,17,18), 130.77 (C-10,15), 116.86, 116.81 (C-5,6,19,20), 114.78 (C-12,13), 71.44 (C-2,23), 70.47 (C-3,22), 70.11, 70.01 (CH_2O), 64.22 (C-1,24), 32.65, 31.34, 30.25, 26.87, 26.56, 23.33, 20.96 (CH_2), 14.34 ppm (CH_3); ^{19}F-NMR (acetone-d_6, 188 MHz): δ = -82.06 (t, ^3J = 9.88 Hz, 3F, CF_3), -115.02 (s, 2F, CH_2CF_2), -122.56 (s, 14F, CF_2), -123.57 (s, 2F, CF_2), -124.30 (s, 2F, CF_2) -127.08 ppm (s, 2F, $\mathbf{CF_2}CF_3$); calculated for $C_{62}H_{77}F_{25}O_8$: C 52.20, H 5.45; found: C 51.76, H 5.74 %.

3-[4´´-(2,3-Dihydroxypropoxy)-5'-eicosyloxy-2'-(5,5,6,6,7,7,8,8,9,9,10,10,11,11,12,12,13,13,14,14,15,15,16,16,16-pentacosafluorhexadecyloxy)-*p*-terphenyl-4-yloxy]-propane-1,2-diol (2):

Synthesized from **2/Ac** (193 mg; 0.13 mmol); purified by crystallisation from THF/MeOH. Yield: 109 mg (60 %), colorless solid; for phase transitions see Tab. 1. ^{1}H-NMR (acetone-d_6, 400 Mhz): δ 7.55 – 7.44 (m, 4H, Ar-*H*), 6.99 – 6.92 (m, 4H, Ar-H), 6.91 (s, 1H, Ar-H), 6.91 (s, 1H, Ar-H), 4.18 – 4.01 (m, 6H, C**H**OH, OCH_2), 3.95 – 3.80 (m, 6H $\mathbf{CH_2}OH$, $O\mathbf{CH_2}CH_2$), 3.80 – 3.71 (m, 2H, $\mathbf{CH_2}OH$), 2.12 – 1.87 (m, 2H, CH_2CF_2), 1.80 – 1.61 (m, 6H, $OCH_2\mathbf{CH_2}$, $\mathbf{CH_2}CH_2CF_2$), 1.41 – 1.14 (m, 34H, $OCH_2CH_2(\mathbf{CH_2})_{17}$), 0.86 (t, *J* = 6.8 Hz, 3H, CH_3); ^{19}F NMR (188 MHz, $CDCl_3$) δ -81.16 (t, *J* = 9.8 Hz), -115.15 (s), -122.08 (s, *J* = 99.9 Hz), -123.38 (s), -124.10 (s), -126.69 (s); HR-ESI-MS: *m/z* [*M*+Cl]$^-$ 1431.4432 (calc. 1431.4589).

3-[4´´-(2,3-Dihydroxypropoxy)-5'-docosyloxy-2'-(5,5,6,6,7,7,8,8,9,9,10,10,11,11,12,12,13,13,14,14,15,15,16,16,16-henicosafluorhexadecyloxy)-*p*-terphenyl-4-yloxy]-propane-1,2-diol (3)

Synthesized from **3/Ac** (115 mg; 0.08 mmol); purified by crystallisation from MeOH. Yield: 74 mg (66 %), colorless solid; for phase transitions see Tab. 1. ^{1}H-NMR ($CDCl_3$, 400 Mhz): δ 7.56 – 7.43 (m, 4H, Ar-H), 6.97 – 6.92 (m, 4H, Ar-H), 6.92 – 6.89 (m, 2H, Ar-H), 4.17 – 4.02

(m, 6H, C**H**OH, OC**H**$_2$), 3.94 – 3.80 (m, 6H C**H**$_2$OH, OC**H**$_2$CH$_2$), 3.80 – 3.71 (m, 2H, C**H**$_2$OH), 2.10 – 1.94 (m, 2H, C**H**$_2$CF$_2$), 1.80 – 1.61 (m, 6H, OCH$_2$C**H**$_2$, C**H**$_2$CH$_2$CF$_2$), 1.38 – 1.10 (m, 38H, OCH$_2$CH$_2$(C**H**$_2$)$_{19}$), 0.86 (t, J = 6.7 Hz, 3H, CH$_3$); ^{19}F NMR (188 MHz, CDCl$_3$): δ -81.15 (t, J = 9.8 Hz), -115.08 (s), -122.12 (s), -122.85 (s), -123.79 (s), -126.61 (s); HR-ESI-MS: *m/z* [*M*+Cl]$^-$ 1359.4767 (calc. 1359.4966).

2. Experimental techniques

2.1 Optical and calorimetric investigations

Phase transitions were determined by polarizing microscopy (Leica DMR XP) in conjunction with a heating stage (FP 82 HT, Mettler) and controller (FP 90, Mettler) and by differential scanning calorimetry (DSC-7, Perkin Elmer) at heating/cooling rates of 10 K min-1 (peak temperatures). Optical investigation was carried out under equilibrium conditions between glass slides which were used without further treatment, sample thickness was ~15 µm. A full wavelength retardation plate was used to determine the sign of birefringence.

2.2 X-ray scattering on powder-like samples

X-ray investigations on powder-like samples were carried out with a Guinier film camera (Huber), samples in glass capillaries (ϕ1 mm) in a temperature-controlled heating stage, quartz-monochromatized CuK$_\alpha$ radiation, 30 to 60 min exposure time, calibration with the powder pattern of Pb(NO$_3$)$_2$.

2.3 Synchrotron X-ray diffraction and electron density reconstruction

High-resolution small-angle powder diffraction experiments were recorded on Beamline I22 at Diamond Light Source and Beamline BL16B1 at Shanghai Synchrotron Radiation Facility, SSRF. Samples were held in evacuated 1 mm capillaries. A modified Linkam hot stage with a thermal stability within 0.2 ºC was used, with a hole for the capillary drilled through the silver heating block and mica windows attached to it on each side. A MarCCD detector was used at both beamlines. q calibration and linearization were verified using several orders of layer reflections from silver behenate and a series of n-alkanes. The measurement of the positions and intensities of the diffraction peaks is carried out using Galactic PeakSolve^{TM} program, where experimental diffractograms are fitted using Gaussian shaped peaks. The diffraction peaks are indexed on the basis of their peak positions, and the lattice parameters and the plane/space groups are subsequently determined. Once the diffraction intensities are measured and the corresponding space group determined, 3D electron density maps can be reconstructed, on the basis of the general formula

$$E(xyz) = \Sigma_{hkl} F(hkl) \exp[i2\pi(hx+ky+lz)] \quad \text{(Eqn. 1)}$$

Here $F(hkl)$ is the structure factor of a diffraction peak with index (hkl). It is normally a complex number and the experimentally observed diffraction intensity

$$I(hkl) = \mathrm{K}\cdot F(hkl)\cdot F^*(hkl) = \mathrm{K}\cdot|F(hkl)|^2 \quad \text{(Eqn. 2)}$$

Here K is a constant related to the sample volume, incident beam intensity etc. In this paper we are only interested in the relative electron densities, hence this constant is simply taken to be 1. Thus the electron density

$$E(xyz) = \Sigma_{hkl} \mathrm{sqrt}[I(hkl)] \exp[i2\pi(hx+ky+lz)+\phi_{hkl}] \quad \text{(Eqn. 3)}$$

for 2D structures $I(hk)$ and Eqn. (4) was used:

$$E(xy) = \Sigma_{hk} \mathrm{sqrt}[I(hk)] \exp[i2\pi(hx+ky) +\phi_{hk}] \quad \text{(Eqn. 4)}$$

As the observed diffraction intensity I(hkl) is only related to the amplitude of the structure factor $|F(hkl)|$, the information about the phase of $F(hkl)$, ϕ_{hkl}, cannot be determined directly from experiment. However, the problem is significantly simplified with most space group

symmetries, where the phase angle ϕ_{hkl} of a given (*hkl*) reflection has limited choices. For example, for centrosymmetric structures ϕ_{hkl} is either 0 or π, but for non-centrosymmetric structures ϕ_{hkl} can be any angle. In this work, (110) peak has a phase angle of ±π/2.
This makes it possible for a trial-and-error approach, where candidate electron density maps are reconstructed for all possible phase combinations, and the "correct" phase combination is then selected on the merit of the maps, helped by prior physical and chemical knowledge of the system. This is especially useful for the study of nanostructures, where normally only a limited number of diffraction peaks are observed.

2.4 Atomic force microscopy

The AFM experiment was performed on a Cypher ES instrument (Asylum - Oxford Instruments) in tapping mode using aluminum-coated tips from Bruker. The AFM scanner was equipped with a heating stage which allows heating of samples in air from ambient to 250°C in a sealed environment. The film was prepared by spin-coating, whereby a solution of compound 3 in toluene was deposited on silicon wafer, and then dried in a vacuum oven.

3. DSC traces and Polarizing microscopy

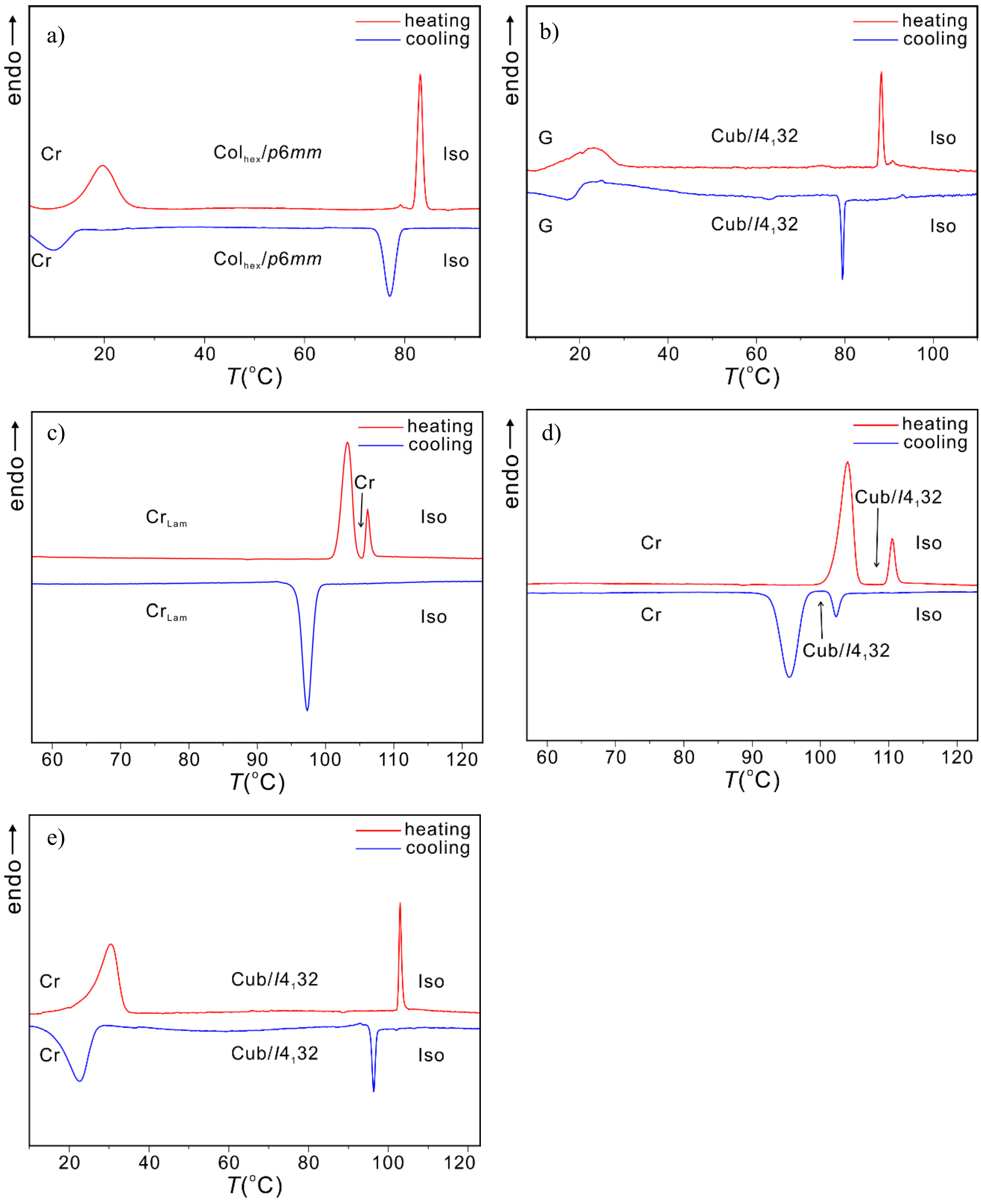

Figure S1. DSC traces upon heating and cooling of compounds: a) **1a**, b) **1b**, c) **1c** (the peak at 105 °C is due to partial crystallization), d) **2**, e) **3**, recorded at 10 K·min^{-1}.

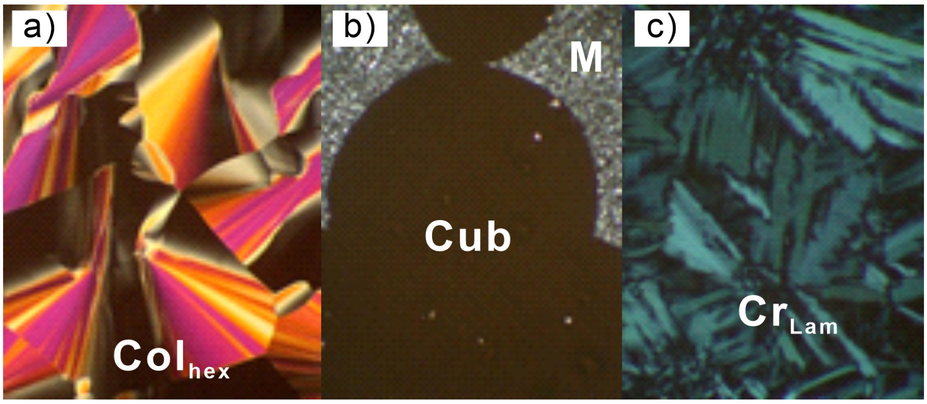

Figure S2. (a - c) Optical textures as observed between under crossed polarizers: of compound **1a** in the Col_{hex} phase at $T = 78$ °C, of the cubic phase (dark areas growing from a metastable lamellar or columnar phase M) of **1b** at $T = 87$ °C and the Cr_{Lam} phase of **1c** at $T =$

4. Additional X-ray and structural data

4.1 Results using laboratory X-ray source

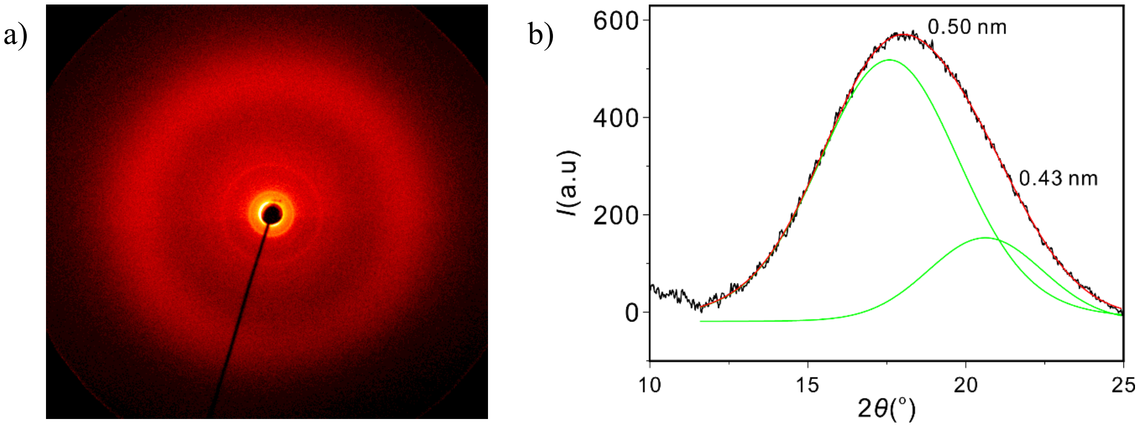

Figure S3. Compound **1a:** (a) X-ray diffraction pattern of the Col_{hex} phase at 70 °C; (b) θ-scan of the wide angle region of the diffraction pattern with the d values for the maxima of the diffuse outer scatterings.

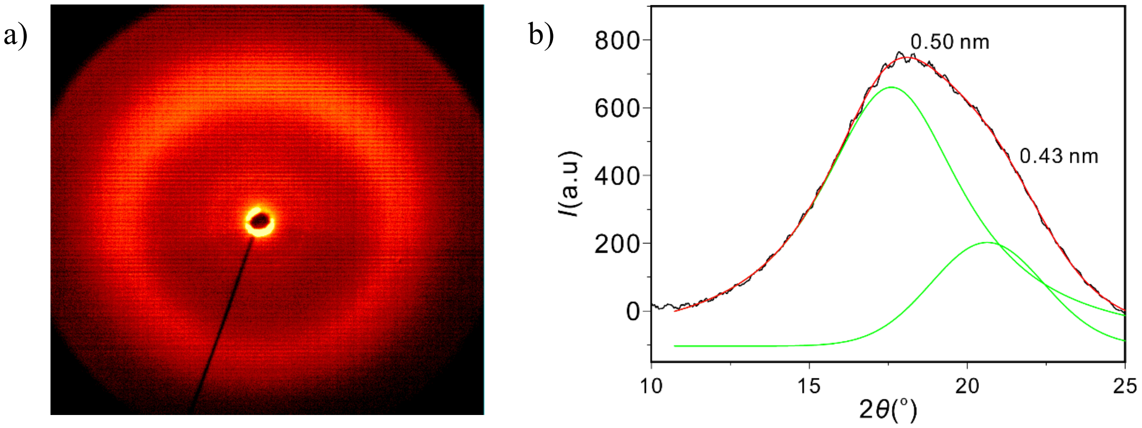

Figure S4. Compound **1b:** (a) X-ray diffraction pattern of the Cub/$I4_132$ phase at 80 °C; (b) θ-scan of the wide angle region of the diffraction pattern with the d values for the maxima of the diffuse outer scatterings.

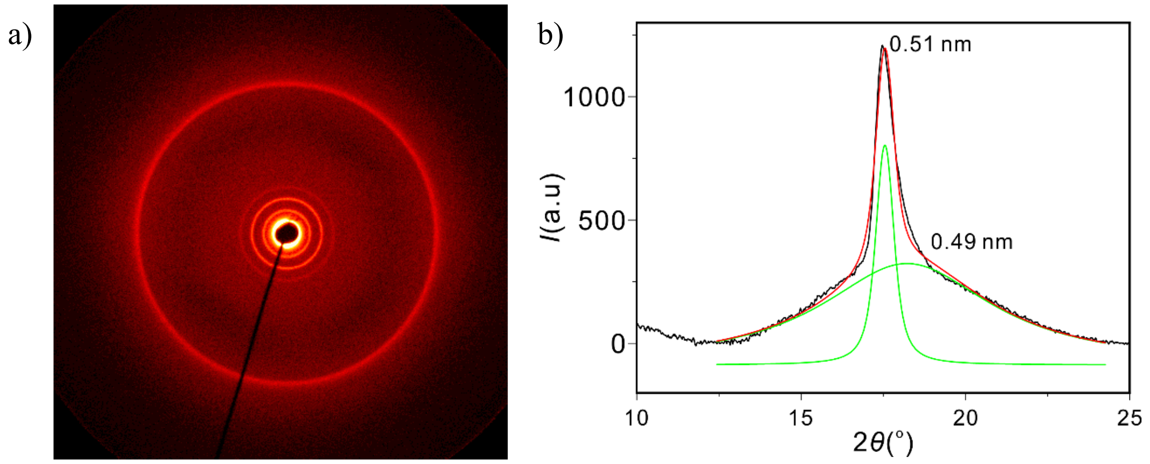

Figure S5. Compound **1c:** (a) X-ray diffraction pattern of the Cr_{Lam} phase at 103 °C; (b) θ-scan of the wide angle region of the diffraction pattern with the *d* value for the maximum of the diffuse outer scattering.

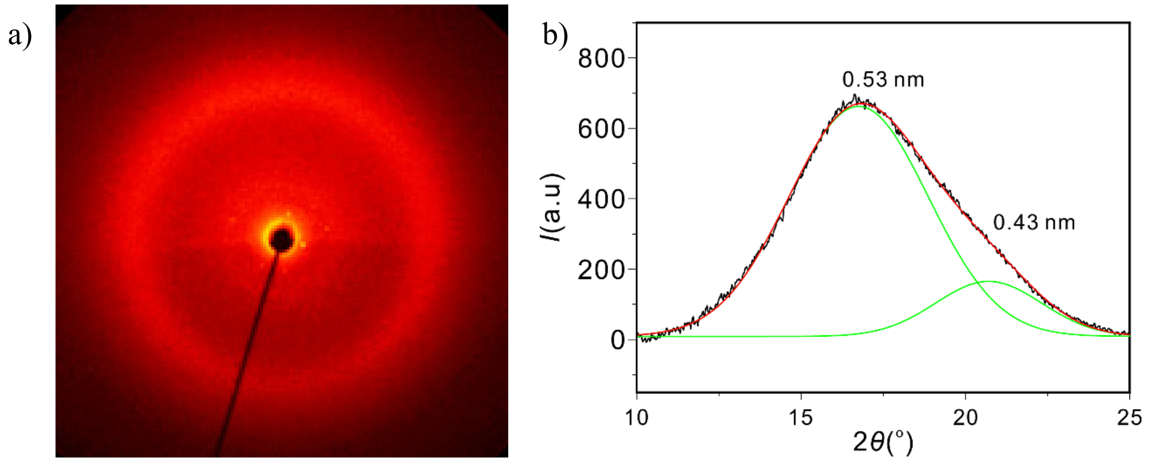

Figure S6. Compound **2:** (a) X-ray diffraction pattern of the Cub/$I4_132$ phase at 105 °C; (b) θ-scan of the wide angle region of the diffraction pattern with the *d* value for the maximum of the diffuse outer scattering.

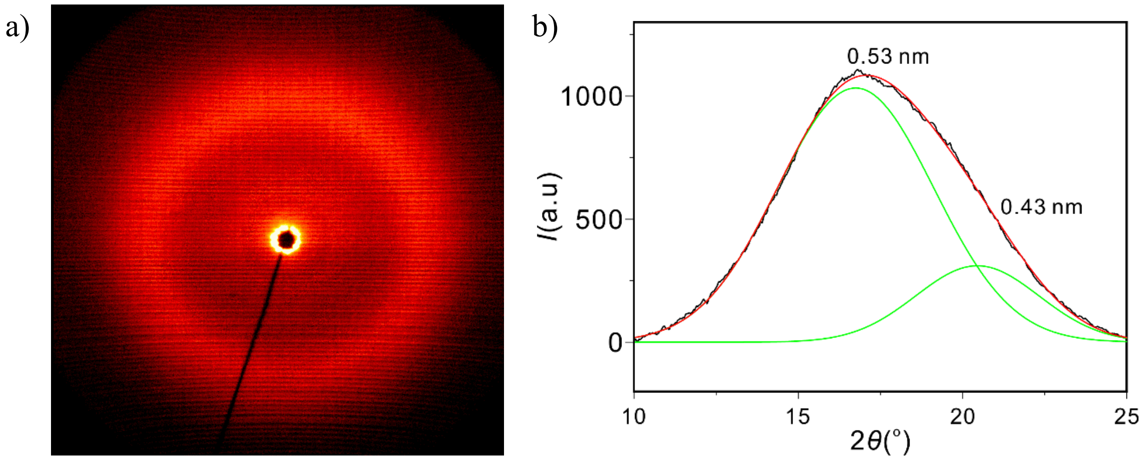

Figure S7. Compound **3:** (a) X-ray diffraction pattern of the Cub/$I4_132$ phase at 80 °C; (b) θ-scan of the wide angle region of the diffraction pattern with the d value for the maximum of the diffuse outer scattering.

4.2 Results using synchrotron sources

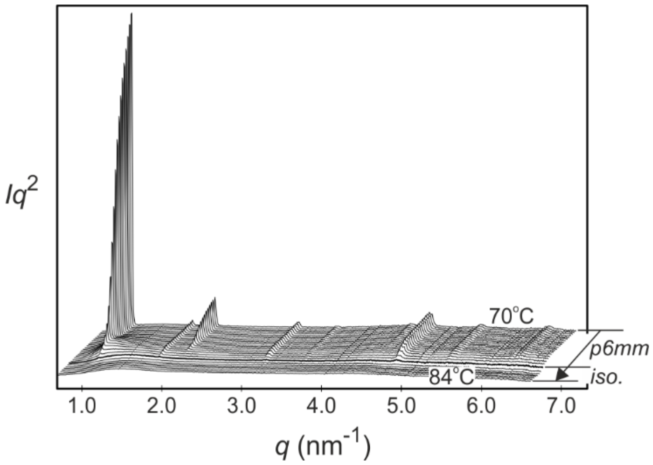

Figure S8. Powder diffractograms of Compound **1a,** continuous heating run from 70 ºC to 84 ºC at the rate of 0.5 ºC/min (back to front), SAXS diffractograms were recorded every 60 seconds.

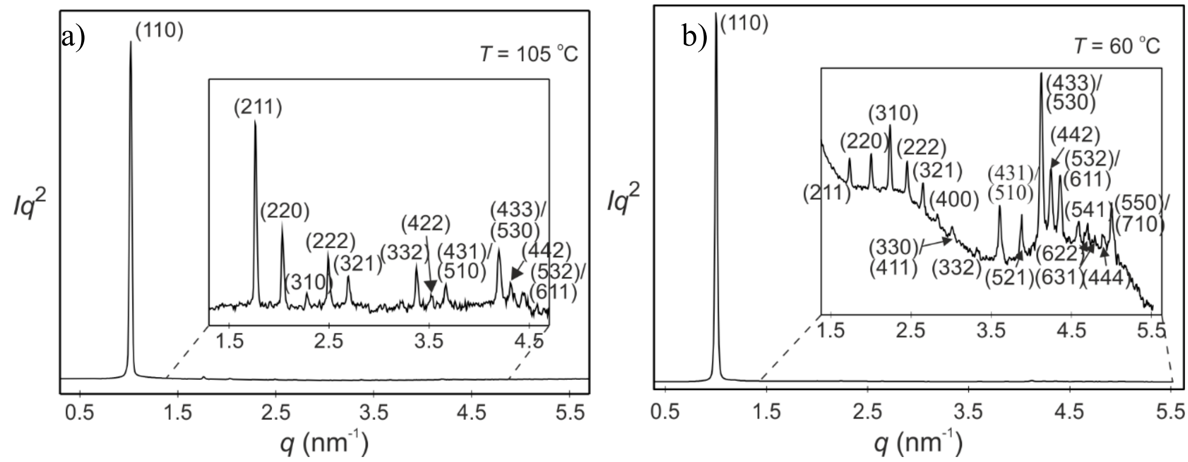

Figure S9. Powder diffractograms of Compounds **2** (a) and **3** (b), recorded at 105 °C and 60 °C respectively.

4.3 Structural data tables

Table S1 Experimental and calculated *d*-spacings of the observed SAXS reflections of the hexagonal phase *p6mm* in Compound **1a** at 70 ºC. All intensities values are Lorentz corrected with correction for multiplicity.

(*hk*)	$d_{obs.}$ –spacing (nm)	$d_{cal.}$ –spacing (nm)	*intensity*	*phase*
(10)	5.97	5.97	100.0	π
(11)	3.45	3.45	2.4	0
(20)	2.99	2.98	11.9	π
(30)	1.99	1.99	3.7	0
(22)	1.72	1.72	1.7	0
(40)	1.49	1.49	1.2	0
(32)	1.37	1.37	1.0	0
(41)	1.30	1.30	6.6	π
(33)	1.15	1.15	3.4	π
(60)	0.99	0.99	0.2	π
a_{hex} = 6.89 nm				

Table S2 Experimental and calculated *d*-spacings of the observed SAXS reflections of the Cubic phase in Compound **1b** at 60ºC. All intensities values are Lorentz corrected with correction for multiplicity.

(*hkl*)	$d_{obs.}$ –spacing (nm)	$d_{cal.}$ –spacing (nm)	*intensity*	*phase*
(110)	6.50	6.50	100.00	π/2
(211)	3.75	3.75	0.02	/
(220)	3.25	3.25	0.06	/
(321)	2.46	2.46	0.05	/
(332)	1.97	1.96	0.03	/
(510)	1.81	1.80	0.15	/
(521)	1.69	1.68	0.02	/
(530)	1.58	1.58	0.43	/
(442)	1.54	1.53	0.20	/
(532)	1.49	1.49	0.07	/
(541)	1.42	1.42	0.01	/
a_{cub} = 9.19 nm				

Table S3 Experimental and calculated *d*-spacings of the observed SAXS reflections of the Lamellar phase in Compound **1c** at 96ºC. All intensities values are Lorentz corrected with correction for multiplicity.

(*hk*)	$d_{obs.}$ –spacing (nm)	$d_{cal.}$ –spacing (nm)	*intensity*	*phase*
(10)	6.32	6.32	100.0	π
(20)	3.15	3.16	4.7	π
(30)	2.10	2.11	11.9	π
(40)	1.57	1.58	4.3	0
d = 6.32 nm				

Table S4 Experimental and calculated *d*-spacings of the observed SAXS reflections of the Cubic phase in Compound **2** at 105ºC. All intensities values are Lorentz and multiplicity corrected.

(*hkl*)	$d_{obs.}$ –spacing (nm)	$d_{cal.}$ –spacing (nm)	*intensity*	*phase*
(110)	6.15	6.15	100.0	π/2
(211)	3.56	3.55	0.3	/
(220)	3.09	3.08	0.3	/
(310)	2.76	2.75	0.01	/
(222)	2.52	2.51	0.3	/
(321)	2.33	2.32	0.03	/
(332)	1.86	1.85	0.1	/
(422)	1.78	1.78	0.02	/
(431)	1.71	1.71	0.04	/
(510)			0.02	/
(433)	1.50	1.49	0.1	/
(530)			0.1	/
(442)	1.45	1.45	0.1	/
(532)	1.41	1.41	0.03	/
(611)			0.01	/
a_{cub} = 8.70 nm				

Table S5 Experimental and calculated *d*-spacings of the observed SAXS reflections of the Cubic phase in Compound **3** at 60ºC. All intensities values are Lorentz and multiplicity corrected.

(*hkl*)	$d_{obs.}$ –spacing (nm)	$d_{cal.}$ –spacing (nm)	*intensity*	*phase*
(110)	6.28	6.28	100.00	π/2
(211)	3.63	3.63	0.02	/
(220)	3.14	3.14	0.06	/
(310)	2.81	2.81	0.06	/
(222)	2.56	2.56	0.09	/
(321)	2.37	2.37	0.01	/
(400)	2.22	2.22	0.06	/
(330)	2.09	2.09	0.01	/
(411)			0.01	/
(332)	1.89	1.89	0.01	/
(431)	1.74	1.74	0.01	/
(510)			0.03	/
(521)	1.62	1.62	0.02	/
(433)	1.52	1.52	0.11	/
(530)			0.11	/
(442)	1.48	1.48	0.11	/
(532)	1.44	1.44	0.02	/
(611)			0.04	/
(541)	1.37	1.37	0.01	/
(622)	1.34	1.34	0.02	/
(631)	1.31	1.31	0.00	/
(444)	1.28	1.28	0.02	/
(550)	1.26	1.26	0.06	/
(710)			0.03	/
a_{cub} = 8.88 nm				

5. Analysis of the two-color Col_{hex} phase of compound 1a

The lattice parameter of the Col_{hex} phase of **1a** corresponds to about three times the molecular length (L_{mol} = 2.3 – 2.6 nm measured between the two terminal polar groups in all-trans conformation). This can be explained by assuming a hexagonal superlattice where each unit cell contains three hexagonal prismatic honeycomb cells, with at least one of them being different from the other two (see Figure 4g in the main text). In principle, in compound **1a** one of the honeycomb cells contains pure alkyl chains while the remaining two may contain a 3:1 R_F-R_H mixture (two-color tiling with *p*6*mm* symmetry). Alternatively the tiling could be three-color, with one cell containing purely R_F chains, one purely R_H chains and the third a 1:1 mixture of R_F and R_H (*p*3*m*1 symmetry). X-ray diffraction alone cannot distinguish between *p*6*mm* and *p*3*m*1 symmetries.[S6] However, it has been shown by mean field theory and Monte Carlo simulation that *p*3*m*1 could only appear far below the temperature of *p*6*mm*,[S7] suggesting that compound **1a** adopts the two-color tiling with *p*6*mm* symmetry. Accordingly, the electron density map reconstructed based on *p*6*mm* (Figure S10) shows a partly segregated two-color tiling composed of a lower-density (alkyl) column and two higher density (mixed) columns.[S7] The aromatic cores make up the walls between the columns with glycerol groups forming the hydrogen bonding networks at cell edges (see Figure 4g in the main text). The number of molecules per unit cell is calculated as n_{cell} = 14.2 (Table S6), which corresponds to a thickness of the walls about 1.5 molecules on average and is in good agreement with the numbers usually obtained for honeycomb phases of X-shaped bolaamphiphiles.[S1a] The number of molecules per unit cell is calculated as n_{cell} = 14.2 (Table S6), which corresponds to a thickness of the walls about 1.5 molecules on average and is in good agreement with the numbers usually obtained for honeycomb phases of X-shaped bolaamphiphiles.[S1a]

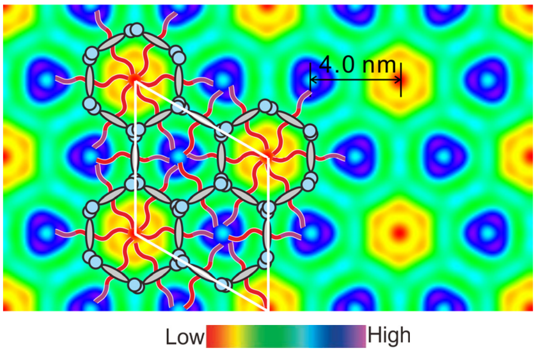

Figure S10. Compound **1a**: Reconstructed electron density map of Col_{hex}/*p*6*mm* phase, some sketched molecules are superposed on the top of the map.

6. Additional electron density maps

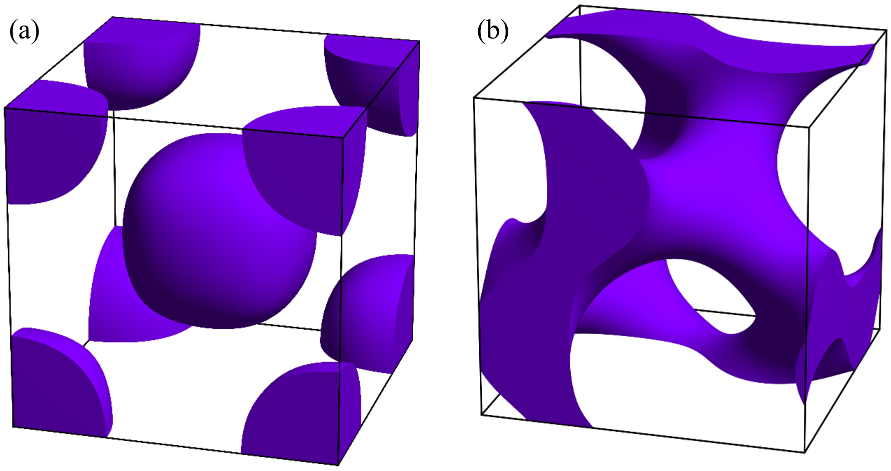

Figure S11. a) Reconstructed electron density map using phase 0 of *Im*3*m* symmetry; b) Reconstructed electron density map using phase π/2 of $I4_132$ symmetry (purple = high electron density region of perfluorinated segments).

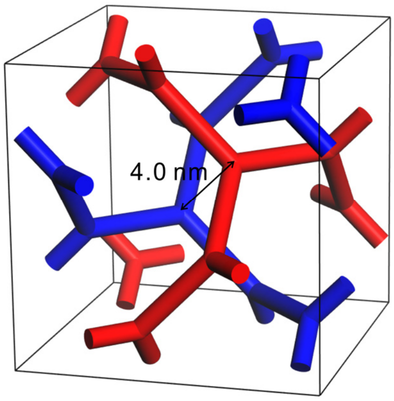

Figure S12. Sketch of alternating double network gyroid phase of compound **1b**.

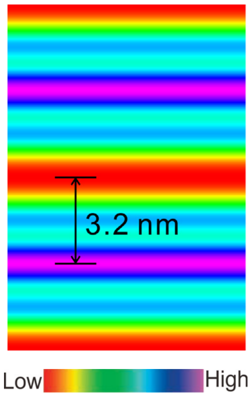

Figure S13. Reconstructed electron density map of Cr_{Lam} phase of compound **1c**.

7. Calculated molecular volume, number of molecules per cell

Table S6. Calculation of the molecular volume (V_{mol}), volume of the hypothetical unit cells (V_{cell}) and number of molecules in these unit cells (n_{cell}) ^{a,b}

Comp.	1a	1b	2	3
a (nm)	6.89	9.19	8.70	8.88
V_{cell} (nm^3)	18.5^a	776.2	658.5	700.2
V_{mol} (nm^3)	1.166	1.226	1.236	1.207
$n_{cell,cryst} = V_{cell} / V_{mol}$	15.9	633.2	532.8	580.2
$n_{cell, liq}$	12.5	497.5	418.6	455.9
n_{cell}	14.2	565.4	475.7	518.1
Minimal Surface S (nm^2)	/	207.2	185.7	193.5
n_{cell} / S	/	2.7	2.6	2.7

^{a} assuming c = 0.45 nm, equal to the distance between π-π stacking; ^{b} V_{cell} = volume of the unit cell defined by the dimensions; V_{mol} = volume for a single molecule as calculated using the crystal volume increments[S8]; $n_{cell, cryst}$ = number of molecules in the unit cell, calculated according to $n_{cell} = V_{cell}/V_{mol}$ (average packing coefficient in the crystal is k = 0.7[S9]; $n_{cell, liq}$ = number of molecules in the unit cell of an isotropic liquid with an average packing coefficient k = 0.55, calculated according to $n_{cell, liq} = 0.55/0.7 \times n_{cell, cryst}$; n_{cell} = in the LC phase estimated as the average of that in the $n_{cell,cryst}$ and $n_{cell,liq}$; Minimal Surface S for $Ia\bar{3}d$ phase is S = 2.4533×a^2.[S10]

8. Face-on view of section through electron density map for comparison with AFM image

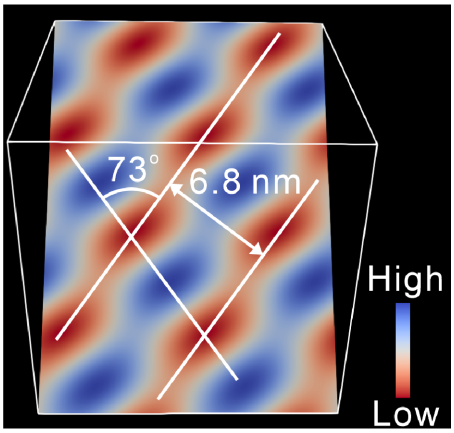

Figure S14. Face-on view of the (110) section through the electron density map of the Cub/$I4_132$ phase of compound **3** (cf. Figure 3b).

9. References

[S1] a) R. Kieffer, M. Prehm, B. Glettner, K. Pelz, U. Baumeister, F. Liu, X. Zeng, G. Ungar, C. Tschierske, *Chem. Commun.* **2008**, 3861-3863; b) F. Liu, R. Kieffer, X. Zeng, K. Pelz, M. Prehm, G. Ungar, C. Tschierske, *Nat. Commun.* **2012**, *3*, 1104.

[S2] B. Glettner, F. Liu, X. Zeng, M. Prehm, U. Baumeister, M. Walker, M. A. Bates, P. Boesecke, G. Ungar, C. Tschierske, *Angew. Chem. Int. Ed.* **2008**, *47*, 9063-9066.

[S3] A. Amini, K. Bates, A. C. Benniston, D. J. Lawrie, E. Soubeyrand-Lenoir, *Tetrahedron Lett.* **2003**, *44*, 8245-8247.

[S4] M. Kölbel, T. Beyersdorff, X. H. Cheng, C. Tschierske, J. Kain, S. Diele, *J. Am. Chem. Soc.* **2001**, *123*, 6809-6818.

[S5] G. Johansson, V. Percec, G. Ungar, J. P. Zhou, *Macromolecules* **1996**, *29*, 646-660.

[S6] H. F. Cheng, Y. X. Li, X. B. Zeng, H. F. Gao, X. H. Cheng, G. Ungar, *Chem. Commun.* **2018**, *54*, 156-159.

[S7] X. Zeng, R. Kieffer, B. Glettner, C. Nürnberger, F. Liu, K. Pelz, M. Prehm, U. Baumeister, H. Hahn, H. Lang, G. A. Gehring, C. H. M. Weber, J. K. Hobbs, C. Tschierske, G. Ungar, *Science* **2011**, *331*, 1302-1306.

[S8] A. Immirzi, B. Perini, *Acta Cryst. A* **1977**, *33*, 216-218.

[S9] A. I. Kitaigorodski, *Akademieverlag*, Berlin, **1979**.

[S10] A. H. Schoen, *NASA Technical Note TN D-5541* **1970**.
